# Endogenous Retroviral Elements Generate Pathologic Neutrophils in Pulmonary Arterial Hypertension

**DOI:** 10.1164/rccm.202102-0446OC

**Published:** 2022-06-13

**Authors:** Shalina Taylor, Sarasa Isobe, Aiqin Cao, Kévin Contrepois, Bérénice A. Benayoun, Lihua Jiang, Lingli Wang, Stavros Melemenidis, Mehmet O. Ozen, Shoichiro Otsuki, Tsutomu Shinohara, Andrew J. Sweatt, Jordan Kaplan, Jan-Renier Moonen, David P. Marciano, Mingxia Gu, Kazuya Miyagawa, Brandon Hayes, Raymond G. Sierra, Christopher J. Kupitz, Patricia A. Del Rosario, Andrew Hsi, A. A. Roger Thompson, Maria E. Ariza, Utkan Demirci, Roham T. Zamanian, Francois Haddad, Mark R. Nicolls, Michael P. Snyder, Marlene Rabinovitch

**Affiliations:** ^1^ Vera Moulton Wall Center for Pulmonary Vascular Diseases,; ^2^ Stanford Cardiovascular Institute,; ^3^ Department of Pediatrics – Cardiology,; ^4^ Department of Genetics,; ^7^ Department of Radiation Oncology,; ^8^ Department of Radiology Canary Center for Cancer Early Detection,; ^9^ Department of Medicine – Pulmonary and Critical Care Medicine, and; ^14^ Department of Medicine – Cardiovascular Medicine, Stanford University, Stanford, California;; ^5^ Leonard Davis School of Gerontology and; ^6^ Department of Molecular and Computational Biology, Dornsife College of Letters, Arts and Sciences, University of Southern California, Los Angeles, California;; ^10^ Linac Coherent Light Source, SLAC National Accelerator Laboratory, Menlo Park, California;; ^11^ Department of Infection, Immunity and Cardiovascular Disease, University of Sheffield, Sheffield, United Kingdom; and; ^12^ Department of Cancer Biology and Genetics and; ^13^ Institute for Behavioral Medicine Research, The Ohio State University Wexner Medical Center, Columbus, Ohio

**Keywords:** extracellular vesicles (EVs), elafin, vinculin, leukocyte elastase, antiviral agents

## Abstract

**Rationale:**

The role of neutrophils and their extracellular vesicles (EVs) in the pathogenesis of pulmonary arterial hypertension is unclear.

**Objectives:**

To relate functional abnormalities in pulmonary arterial hypertension neutrophils and their EVs to mechanisms uncovered by proteomic and transcriptomic profiling.

**Methods:**

Production of elastase, release of extracellular traps, adhesion, and migration were assessed in neutrophils from patients with pulmonary arterial hypertension and control subjects. Proteomic analyses were applied to explain functional perturbations, and transcriptomic data were used to find underlying mechanisms. CD66b-specific neutrophil EVs were isolated from plasma of patients with pulmonary arterial hypertension, and we determined whether they produce pulmonary hypertension in mice.

**Measurements and Main Results:**

Neutrophils from patients with pulmonary arterial hypertension produce and release increased neutrophil elastase, associated with enhanced extracellular traps. They exhibit reduced migration and increased adhesion attributed to elevated β1-integrin and vinculin identified by proteomic analysis and previously linked to an antiviral response. This was substantiated by a transcriptomic IFN signature that we related to an increase in human endogenous retrovirus K envelope protein. Transfection of human endogenous retrovirus K envelope in a neutrophil cell line (HL-60) increases neutrophil elastase and IFN genes, whereas vinculin is increased by human endogenous retrovirus K deoxyuridine triphosphate diphosphatase that is elevated in patient plasma. Neutrophil EVs from patient plasma contain increased neutrophil elastase and human endogenous retrovirus K envelope and induce pulmonary hypertension in mice, mitigated by elafin, an elastase inhibitor.

**Conclusions:**

Elevated human endogenous retroviral elements and elastase link a neutrophil innate immune response to pulmonary arterial hypertension.

**At a Glance Commentary**

**Scientific Knowledge on the Subject**

Pulmonary arterial hypertension (PAH) is a disease characterized by occlusion of distal pulmonary arteries resulting in increased resistance to flow and culminating in heart failure. The appearance of fragmented elastic laminae in pulmonary arteries from patients with PAH suggested the presence of heightened elastase activity in the vessel wall that was identified as neutrophil elastase in human cells and in experimental animals with pulmonary hypertension.

**What This Study Adds to the Field**

We describe a novel phenotype of neutrophils related to an increase in *1*) production and release of elastase, *2*) generation of neutrophil extracellular traps, and *3*) adhesion mediated by vinculin that directly contributes to the pathogenesis of PAH. This phenotype results from a cell-autonomous and non–cell-autonomous increase in two human endogenous retrovirus K family retroviral elements.

Pulmonary arterial hypertension (PAH) is a disease characterized by occlusion of distal pulmonary arteries resulting in increased resistance to flow and culminating in heart failure. Current treatments improve quality of life and survival primarily by dilating pulmonary arteries, but these agents do not address the mechanism underlying the progressive pulmonary vascular pathology (reviewed in [1]). The appearance of fragmented elastic laminae in pulmonary arteries from patients with PAH suggested the presence of heightened elastase activity in the vessel wall (2). Increased pulmonary arterial smooth muscle cell (SMC) elastase activity was identified as neutrophil elastase (NE) in human cells and in experimental animals with pulmonary hypertension (3). A consequence of heightened NE activity is degradation of elastin, leading to increased vascular stiffness (4), generation of elastin peptides that are highly chemotactic to monocytes (5), and release of growth factors from the extracellular matrix (6) that activate growth factor receptors (7), resulting in SMC proliferation (8). Inhibition of NE with synthetic inhibitors (9) or with the recombinant human elastase inhibitor elafin (10) is effective in preventing and reversing pulmonary hypertension in animal models and promoting regression of pulmonary arterial pathology in human organ cultures (10).

Neutrophils are major sources of NE; increased release of NE is observed in PAH neutrophils (11), and circulating NE concentrations are elevated in plasma from patients across all PAH subtypes (12, 13) and are associated with worsening clinical severity and deficiency in the elastase inhibitor elafin (13). Moreover, the neutrophil-to-lymphocyte ratio is increased in PAH and also correlates with clinical deterioration judged by New York Heart Association functional class and event-free survival (14). Other alterations in neutrophil functions in response to injury include propensity for neutrophil extracellular trap (NET) formation (15) and alterations in adhesion and migration (16).

NE is critical for NET formation, and the enzyme remains proteolytically active when bound to NETs, thus contributing to tissue damage (17). Elevated markers of NET formation are observed in end-stage plexiform lesions in patients with PAH (12). In addition to local effects, NE exerts remote consequences by being packaged and transported in extracellular vesicles (EVs). BAL from patients with chronic obstructive pulmonary disease (COPD) contains EVs enriched with NE that caused alveolar destruction when injected intratracheally in mice (18).

To understand their contribution to the pathogenesis of PAH, we isolated circulating neutrophils from patients with PAH versus healthy control subjects and first assessed NE production and release, NET formation, and adhesion and migration, including transendothelial migration. We performed proteomic and transcriptomic profiling to identify the mechanism underlying the abnormalities observed. Our results indicated a novel antiviral response in PAH neutrophils that explained the PAH neutrophil phenotype. The mechanism accounting for increased elastase was related to an elevation in the human endogenous retrovirus (HERV)-K envelope in neutrophils, and the mechanism explaining increased vinculin (VCL)-mediated adhesion could be attributed to elevated circulating concentrations of HERV-K deoxyuridine triphosphate diphosphatase (dUTPase). We showed that PAH neutrophil EVs contain elevated NE as well as the HERV-K envelope and that they induce pulmonary hypertension in mice, except when pretreated with elafin (19). Taken together, our studies can explain how neutrophil dysfunction contributes to PAH and can be targeted therapeutically. Some of the results of these studies were previously reported in the form of abstracts (20–22).

## Methods

An Expanded Methods section is available in the online supplement.

### PAH Patient and Control Samples

Whole blood from patients with PAH was obtained from the Stanford PAH biobank, and blood from healthy volunteers was obtained from the Stanford Biobank and the Stanford Blood Center. Pulmonary arterial endothelial cells (PAECs) were obtained through the Pulmonary Hypertension Breakthrough Initiative (*see* Tables E1–E4 in the online supplement).

### Reagents

*See* Table E6.

### Neutrophil Isolation

Human neutrophils were purified from peripheral blood using Miltenyi Biotec MACSxpress Neutrophil isolation kit.

### HL-60 and dHL-60 Cell Culture

HL-60 cells were cultured in RPMI 1640 complete media and treated with 1.3% DMSO for 6 to 7 days for differentiation to dHL-60 cells.

### HERV-K dUTPase Protein Purification

Recombinant HERV-K dUTPase protein was provided by M.E.A. See the methods in the online supplement for additional information.

### PAEC Culture

PAECs were harvested from explanted lungs of patients with PAH removed at the time of lung transplant or from unused donor lungs (controls) and were cultured in endothelial cell complete medium.

### NE Activity

NE activity was measured using EnzChek Elastase Assay Kit (Thermo Fisher Scientific).

### NET Formation

Neutrophils adherent to poly-d-lysine–coated coverslips were treated with phorbol myristate acetate (PMA) with vehicle (phosphate-buffered saline [PBS]) or elafin for 60 minutes. Cells were washed with PBS and then stained with cell-impermeable SYTOX Green and for H3 citrullinated and NE as described in the Expanded Methods section of the online supplement.

### Migration (Chemokinesis)

Neutrophils adherent to fibronectin were treated with 100 ng/mL of IL-8, and cell migration was observed in real time using a confocal microscope.

### Transmigration and Transendothelial Migration

Transmigration and transendothelial migration assays were performed in a modified Boyden chamber using the Corning FluoroBlok 24-well plate.

### Adhesion

Neutrophils were incubated on fibronectin-coated slides or on a monolayer of PAECs at 37°C for 5 minutes, washed with PBS, then imaged under light microscopy.

### Flow Cytometry

Neutrophils were stained for Alexa Fluor 700–conjugated integrin-β2 (ITGB2), and data were acquired using a Beckton Dickenson LSR II flow cytometer and analyzed using FlowJo.

### Immunofluorescence Staining

Adherent neutrophils were fixed with 4% paraformaldehyde and treated with HERV-K envelope antibody, followed by Alexa Fluor 488–conjugated secondary antibody and DAPI staining.

### Transmission Electron Microscopy

Pooled EV samples were visualized using a JEOL JEM-1400 120-kV electron microscope.

### Western Immunoblot Analysis

See the Expanded Methods section in the online supplement.

### RT-qPCR

qPCR was performed using Powerup SYBR Green PCR Master Mix (Applied Biosystems).

### Primers

Primer sequences are listed in Table E7.

### Plasmid Transfection

HL-60 cells were transfected with HERV-K envelope vector or enhanced green fluorescent protein control vector (VectorBuilder) using HL-60 Cell Avalanche Transfection Reagent (EZ Biosystems).

### EV Isolation from Plasma and CD66b Pulldown

EVs were isolated from plasma using the EV Total Isolation Chip (ExoTIC). ExoTIC-harvested EVs were further purified using Dynabeads conjugated to CD66b/CEACAM-8 (carcinoembryonic antigen–related cell adhesion molecule 8).

### Untargeted Proteomics by Liquid Chromatography–Mass Spectrometry

Proteomic analysis used the liquid chromatography system directly coupled inline with an Orbitrap Fusion Lumos Mass Spectrometer.

### RNA-Sequencing Analysis

Total RNA was prepared using the Takara Bio SMARTer Stranded Total RNA-Seq Kit version 2 Pico Input Mammalian kit and sequenced using the HiSeq 2500 sequencer.

### Mouse Model for the Induction of Pulmonary Hypertension by Neutrophil EVs

Adult male mice (8 wk of age) were administered tail vein injections twice weekly for 5 weeks of *1*) PBS vehicle, *2*) pooled EVs of healthy donor controls, *3*) pooled PAH EVs, or *4*) pooled elafin-treated PAH EVs. Cardiac function, right ventricular systolic pressure, right ventricular hypertrophy, and pulmonary vascular morphometry were assessed. The first cohort consisted of the first three groups with *n* = 3 in each group, and the second cohort added the fourth group with *n* = 6 in each group.

### Statistical Analysis

Bars represent the mean ± SEM, and *n* is the number of control subject or patient cells studied. Statistical significance was determined by one-way ANOVA followed by Dunnett’s multiple comparison test, or it was determined by two-sided unpaired *t* test. Data with a sample size *n* < 8 were analyzed using the Mann-Whitney nonparametric test. Samples with counts of 3 are confirmatory of other assays or represent technical replicates from cell lines or pooled samples. Pathway enrichment analysis was conducted using the Integrated Molecular Pathway-Level Analysis (IMPaLA) webtool.

### Data Availability

RNA-sequencing (RNA-seq) data were deposited in the National Center for Biotechnology Information Sequence Read Archive with accession number PRJNA822411. Proteomic data are available via ProteomeXchange with identifier PXD023134. Code related to RNA-seq data analysis is available on the Benayoun lab GitHub website.

## Results

### Increased NE Protein and NE-mediated NETosis in PAH Neutrophils

**Figure 1.**
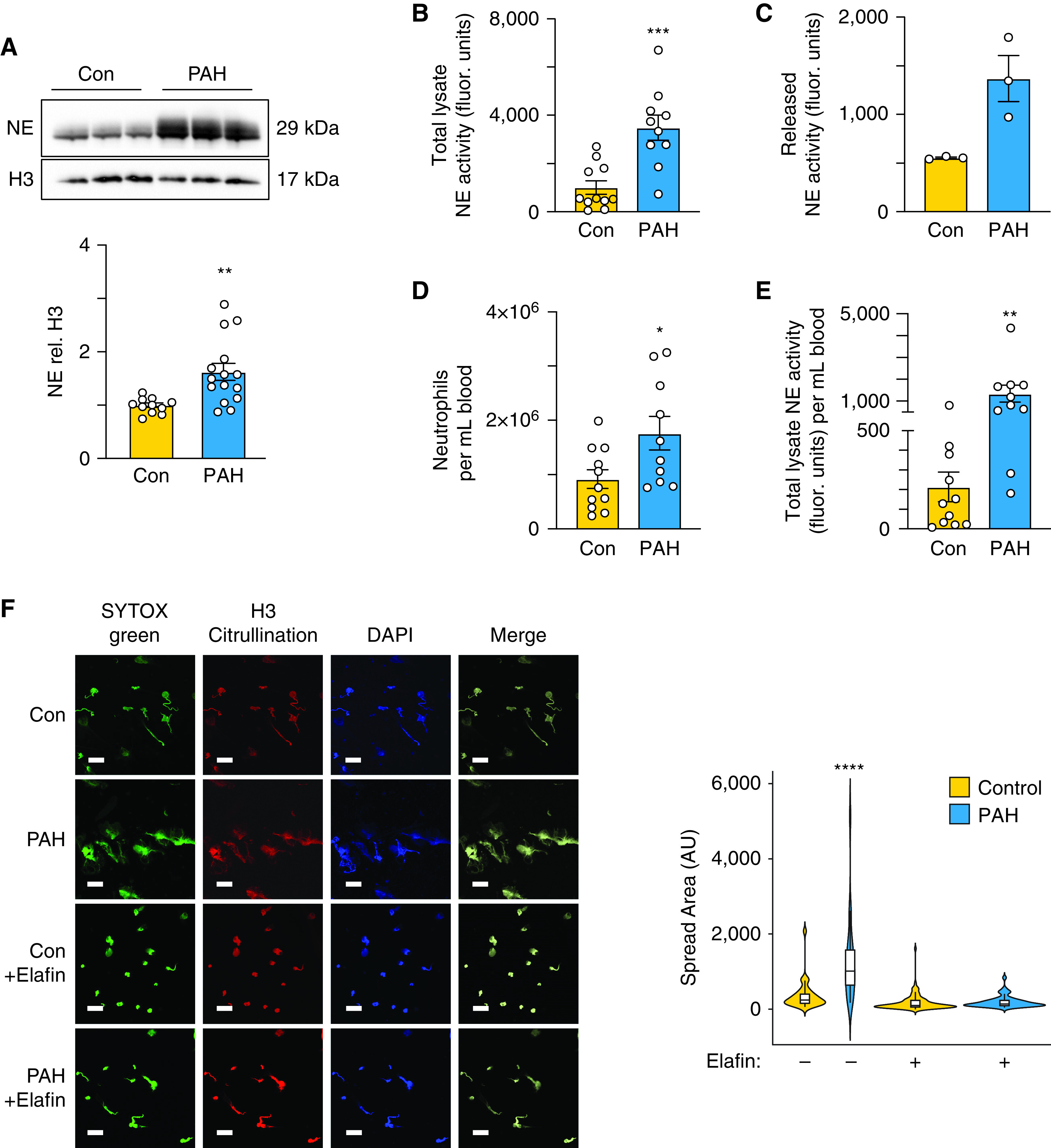
Increased neutrophil elastase (NE) protein and NE-mediated NETosis in pulmonary arterial hypertension (PAH) neutrophils. Neutrophils were isolated from peripheral blood of healthy donor controls (Con) and patients with PAH as described in the Methods section. (*A*) Representative western immunoblot and quantification below, displaying NE relative to histone H3 (H3) in PAH versus Con neutrophils (*n* = 8 Con and *n* = 12 PAH). (*B*) NE activity in neutrophil cell lysates as determined by production of BODIPY FL–labeled fluorescent elastin fragments from self-quenching BODIPY FL–conjugated bovine neck ligament elastin (*n* = 11 Con and *n* = 10 PAH). (*C*) NE activity in the extracellular supernatant 2 hours after IL-8 stimulation of neutrophils (*n* = 3). In *A*–*E*, 5 × 10^6^ cells were used for each assay. (*D*) Number of neutrophils per milliliter of blood in Con and PAH, determined using a Millipore scepter cell counter (*n* = 11 Con and *n* = 10 PAH). (*E*) Total NE activity calculated per 1 mL of blood. NE activity measured in *B* relative to neutrophils counted per *D* to yield NE per milliliter of blood. (*F*) Neutrophil extracellular traps (NETs) were visualized via H3 citrullination (red) and SYTOX Green and quantified by SYTOX Green fluorescence 60 minutes after phorbol myristate acetate stimulation and treatment with vehicle (Veh) or the NE inhibitor elafin (1 μg/mL). The nuclei were counterstained with DAPI (blue). Scale bar, 40 μm. Violin plots represent the variable distribution of four representative biological replicates (*n* = 24–104 cells). In *A*–*E*, bars represent mean ± SEM. *P < 0.05, **P < 0.01, ***P < 0.001 by unpaired Student’s *t* test (*A*, *B*, *D*, *E*). In *F*, for the violin plots, ****P < 0.0001 by one-way ANOVA followed by Dunnett’s *post hoc* test. AU = arbitrary units.

Neutrophils isolated from the blood of patients with PAH and control subjects were confirmed to be >95% pure, both by morphology and by fluorescence-activated cell sorter (FACS) analysis of CD66b and CD16, as shown in Figure E1A and described in the Expanded Methods section of the online supplement. Demographics and other data related to the cohorts used in each of the experiments are found in Tables E1–E4 in the online supplement. Although we requested samples from patients with idiopathic PAH, a small proportion (3 of 68) were later reclassified as having drug- and toxin-related group 1 PAH (23), and 5 of 68 had some cardiopulmonary comorbidities not believed to be causing the PAH. We observed an almost twofold increase in concentrations of NE protein in PAH neutrophils when compared with those of control subjects as assessed by western immunoblot analysis (Figure 1A). A comparable increase in NE activity was established using a fluorescently labeled (DQ) elastin substrate (Figure 1B), and the release of NE activity observed in response to IL-8 stimulation (Figure 1C and time course in Figure E1B) is consistent with these observations. More than two-thirds of elastase activity measured by DQ elastin could be attributed to NE, assessed using a selective inhibitor, N-methoxysuccinyl-Ala-Ala-Pro-Val-chloromethyl ketone. There also appeared to be an elevation in non-NE elastolytic activity in PAH neutrophils (Figures E1C and E1D); however, this did not reflect an overall increase in proteolytic activity, because concentrations of myeloperoxidase were similar in control and PAH neutrophils (Figure E1E).

Elevated NE activity was normalized to neutrophil counts. These were also increased in this cohort of patients with PAH compared with control subjects (Figure 1D), resulting in higher NE activity per milliliter of blood (Figure 1E). NE contributes to the release of NETs by translocating from granules to the nucleus to cleave histones, causing chromatin decondensation and release (15). NETosis incurs tissue damage, including endothelial cell injury (17), through the exteriorization of chromatin accompanied by NE. Using SYTOX Green and citrullinated histone 3 staining of the spread area of exteriorized DNA, we showed heightened release of NETs upon PMA stimulation of PAH versus control neutrophils that was inhibited by the NE inhibitor elafin (10) (Figure 1F). We also showed increased NE exteriorized on NETs from patients with PAH (Figure E1E). It is interesting that the low degrees of NET formation in the control cells are not completely abolished by elafin. This could reflect the low sensitivity of the assay at those degrees of NET formation or that a mechanism for NET formation is unrelated to elastase in the control cells (24) or that elafin does not penetrate control cells.

### PAH Neutrophils Exhibit Increased Adhesion and Reduced Migration

**Figure 2.**
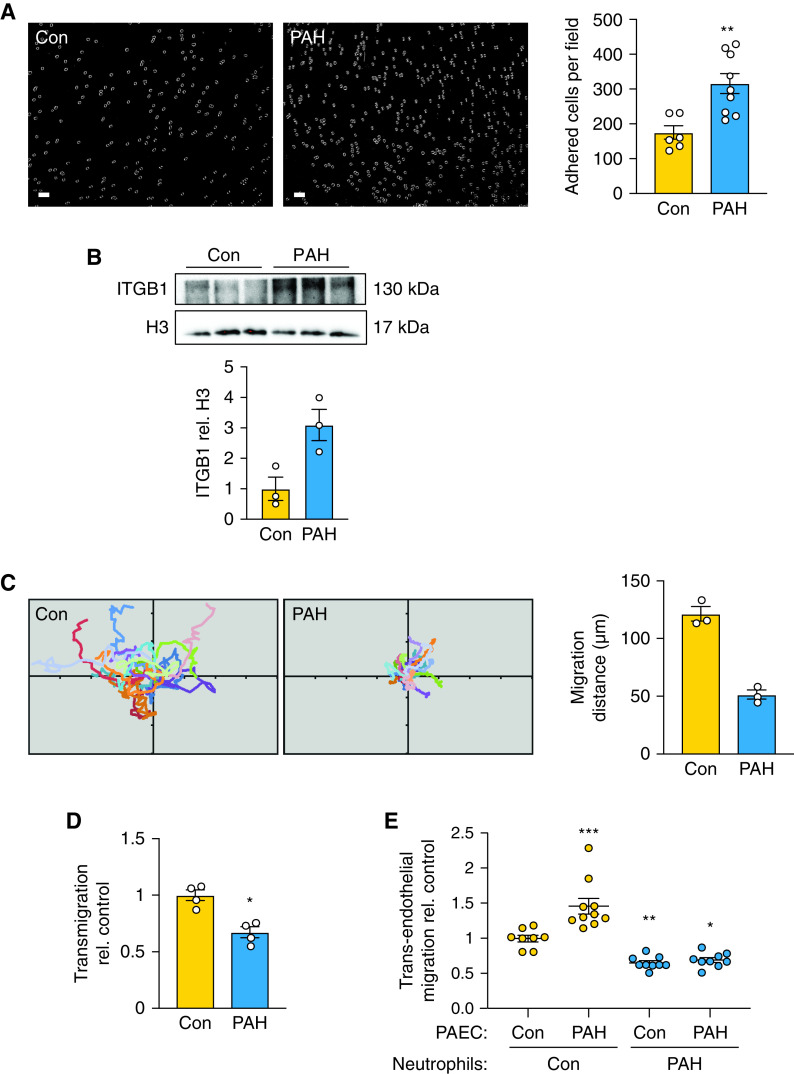
Pulmonary arterial hypertension (PAH) neutrophils exhibit increased adhesion and reduced migration. (*A*) Neutrophils were incubated on fibronectin-coated coverslips for 5 minutes, and the mean number of adherent cells was assessed from three to five randomly selected visual fields. Representative image of adherent neutrophils and quantification on the right (*n* = 6 control [Con] and *n* = 9 PAH). Scale bar, 40 μm. (*B*) Representative western immunoblot and quantification below of integrin-β1 (ITGB1) in PAH versus Con neutrophils (*n* = 3). (*C*) Neutrophils were allowed to adhere to fibronectin-coated coverslips for 5 minutes and then stimulated with 100 ng/mL IL-8 in RPMI 1640 for 30 minutes. Migration of PAH versus Con neutrophils is illustrated as spider plots, and distance migrated was quantified (on the right) using MTrackJ (ImageJ). Quantification was performed for at least 10 migratory cells per field (*n* = 3 fields). (*D*) Calcein-AM–labeled PAH versus Con neutrophils were plated on fibronectin-coated Transwell chambers and then stimulated with 100 ng/mL IL-8. Transmigration was assessed after 60 minutes of stimulation (*n* = 4). (*E*) Calcein-AM–labeled PAH versus Con neutrophils were incubated on Con or PAH pulmonary arterial endothelial cell (PAEC) monolayers grown to confluence in Transwell chambers. Transendothelial migration of PAH versus Con neutrophils across Con or PAH PAECs 60 minutes after IL-8 (100 ng/mL) stimulation (*n* = 8–12). In *A*–*D*, bars represent mean ± SEM. *P < 0.05, **P < 0.01, by Mann-Whitney test (*A*, *D*). In *E*, range represents mean ± SEM. *P < 0.05, **P < 0.01, ***P < 0.001 by one-way ANOVA followed by Dunnett’s *post hoc* test.

Increased NE production and NETosis suggested that other neutrophil functions could be altered or exaggerated in response to inflammatory stimuli. The functions considered were adhesion, migration, and transendothelial migration. We observed heightened adhesion to a fibronectin substrate in PAH versus control neutrophils (Figure 2A) that we related to an increase in β1-integrin (ITGB1; Figure 2B). This was consistent with reduced chemokinesis of PAH versus control neutrophils as assessed by confocal microscopic live-cell imaging of total distance moved in response to IL-8 stimulation (Figure 2C, Video 1) and longitudinal distance migrated across the fibronectin substrate (Figure 2D) and with formylmethionylleucylphenylalanine stimulation (Figure E2A), suggesting that impaired migration appeared to be independent of a specific receptor-activated pathway. IL-8 stimulation of neutrophil migration across a monolayer of PAH or control PAECs was next assessed. Although control neutrophils exhibited increased migration across PAH versus control PAECs, PAH neutrophils displayed impaired migration across both control and PAH PAECs (Figure 2E), suggesting that the impaired neutrophil migration was not compensated by the PAH PAECs. This also suggests that although neutrophils may be attracted to the vessel wall, their increased adhesion to the endothelium, shown in Figure E2B, may lead to NET formation and to failed emigration from the tissue.

### Proteomic Analysis Links Increased VCL to PAH Neutrophil Dysfunction

**Figure 3.**
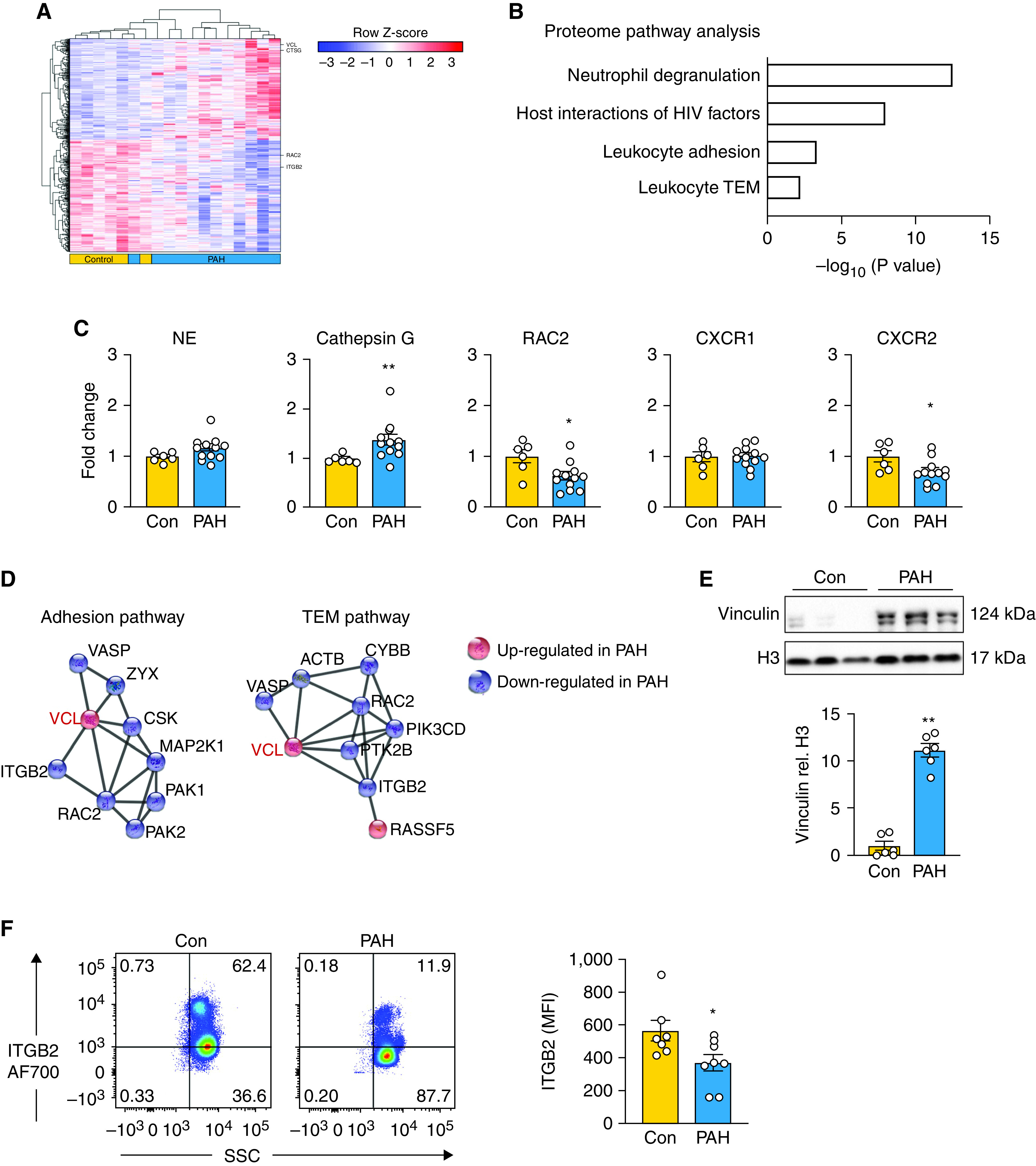
Proteomic analysis links increased vinculin to pulmonary arterial hypertension (PAH) neutrophil dysfunction. (*A*) Unbiased proteomic analysis of isolated PAH versus control (Con) neutrophils by mass spectrometry. Heatmap depicts unsupervised hierarchical clustering of 483 differentially expressed proteins between the two groups (adjusted *P* < 0.10; *n* = 6 Con and *n* = 12 PAH). (*B*) Integrated Molecular Pathway-Level Analysis reflecting significant differentially expressed proteins in PAH versus Con neutrophils consistent with functional abnormalities identified in Figures 1 and 2. (*C*) Fold change in neutrophil elastase (NE), cathepsin G, RAC2, CXCR1, and CXCR2 from proteomic analysis comparing PAH versus Con neutrophils (*n* = 6 Con and *n* = 12 PAH). (*D*) STRING analysis of significant proteins in the adhesion and transendothelial migration (TEM) pathways depicting proteins up- or downregulated in PAH versus Con. (*E*) Representative western immunoblot and quantification below validating the upregulation of vinculin in PAH versus Con neutrophils (*n* = 6). (*F*) Validation and quantification of ITGB2 expression in neutrophils from Con or PAH by fluorescence-activated cell sorter analysis. MFI = mean fluorescence intensity; SSC = side scatter. Representative scatterplots on the left with quantification on the right (*n* = 7 Con and *n* = 8 PAH). In *C*, *E*, and *F*, bars represent mean ± SEM. *P < 0.05, **P < 0.01 by Mann-Whitney test.

To investigate a mechanism that could explain the increase in NE activity and neutrophil adhesion and the decrease in migration, we performed protein expression profiling by high-resolution mass spectrometry nontargeted proteomics on unstimulated cells. Principal component analysis of the proteome revealed some overlap between PAH and control neutrophils (Figure E3A), but 483 differentially regulated proteins were identified with a false discovery rate (FDR) of 10% (Figure 3B). Pathway enrichment analysis from IMPaLA revealed proteins related to neutrophil degranulation, host interactions with HIV factors, adhesion, and transendothelial migration (Figures 3B and E3B). Although we documented an increase in elastase protein by Western immunoblot analysis and activity by substrate assay, the increase in NE evident by proteomic analysis gave a *P* value of 0.04 but an FDR of 11% (Figure 3C) and would have been missed with the 10% FDR. This result reinforces the limitations of high-throughput analyses. Cathepsin G, another serine protease present in azurophilic granules, was increased with a *P* value of 0.006 and an FDR of 6% (Figure 3C). No significant differences were evident in other azurophilic proteases, such as myeloperoxidase and azurocidin (Figure E3C).

Proteomic analysis also revealed reduced RAC2 (Ras-related Rho GTPase) (*P* = 0.03; FDR, 9%) and C-X-C motif chemokine receptor 2 (CXCR2) (*P* = 0.04; FDR, 11%; Figure 3C) in PAH versus control samples. RAC2 is an important mediator of neutrophil chemotaxis (25), and IL-8 induces a migratory response through CXCR2, as reviewed elsewhere (26). The reduction in RAC2 and CXCR2 could therefore contribute to decreased PAH neutrophil migration. CXCR1 is involved in neutrophil degranulation (27) but was unchanged in the PAH versus control samples. STRING analysis was applied to find central protein nodes that could reveal key regulators in the adhesion and transendothelial migration pathways. VCL was identified as a central node in PAH samples that was linked to a reduction in ITGB2, also known to play an integral role in adhesion and migration (28) (Figure 3D). We confirmed the increase in VCL in PAH versus control neutrophils by western immunoblot analysis (Figure 3E), and we confirmed the decrease in ITGB2 by FACS (Figure 3F). The increase in adhesion could be explained by heightened VCL, because VCL-knockout neutrophils show reduced adhesion (29). Moreover, fibronectin was used as the substrate for adhesion, and a strong VCL–fibronectin complex was previously reported (30) that could be explained by the increase in ITGB1 shown in Figure 2B. The reduction in ITGB2, like that of RAC2 and CXCR2, could explain reduced migration (31).

### Transcriptomic Analysis Reveals an Antiviral Signature in PAH Neutrophils

**Figure 4.**
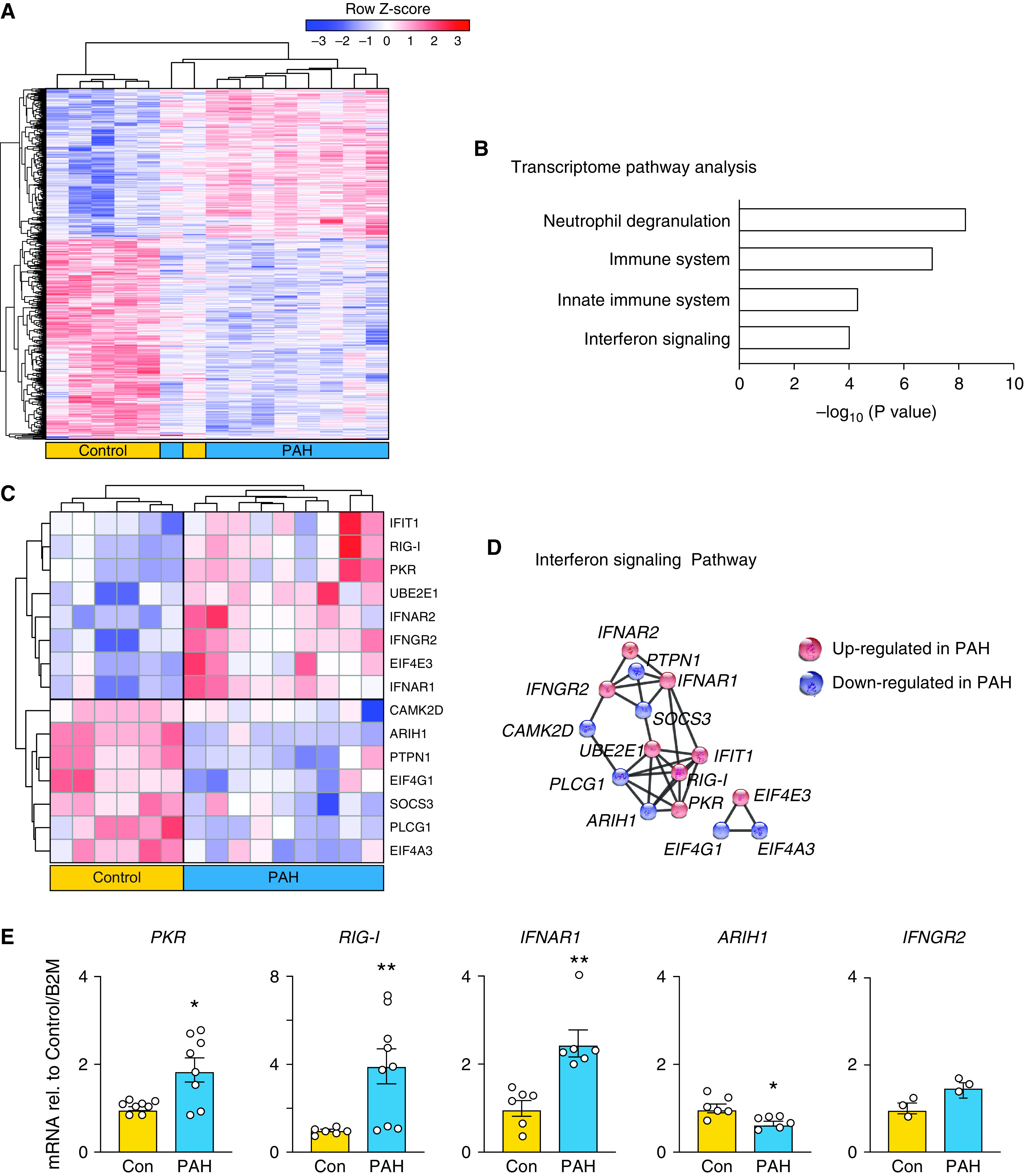
Transcriptomic analysis reveals an antiviral signature in pulmonary arterial hypertension (PAH) neutrophils. (*A*) Unbiased transcriptomic analysis was performed on neutrophils isolated from PAH versus control (Con) patients. Heatmap depicts unsupervised hierarchical clustering of 1,565 differentially expressed genes between the two groups (adjusted *P* < 0.05; *n* = 6 Con and *n* = 9 PAH). (*B*) Integrated Molecular Pathway-Level Analysis (IMPaLA) of significant genes in PAH versus Con neutrophils. (*C*) Heatmap of significant differentially regulated genes in the IFN pathway derived from IMPaLA. (*D*) STRING analysis of significantly changed genes in the IFN pathway depicting genes that are up- or downregulated with key central nodes in PAH versus Con. (*E*) mRNA expression by PCR validating the changes in *PKR*, *RIG-I*, *IFNAR1*, *ARIH1*, and *IFNGR2* observed in neutrophils isolated from Con or patients with PAH. Bars represent mean ± SEM. *PKR* (*n* = 8), *IFNR1* and *ARIH1* (*n* = 6), *RIG-I* (*n* = 6 Con and *n* = 9 PAH), and *INFNGR*2 (*n* = 3). *P < 0.05, **P < 0.01 by Mann-Whitney test.

To elucidate mechanisms that could explain why NE and VCL are increased in PAH versus control neutrophils, we conducted transcriptomic analyses using RNA-seq. The principal component analysis of the transcriptome revealed better separation of unstimulated PAH and control neutrophils when compared with the proteomic analysis (Figure E4A). Using an FDR <5%, we identified 1,483 differentially expressed genes as displayed in the heat map (Figure 4A). By using pathway enrichment analysis from IMPaLA, the top enriched pathway was related to neutrophil degranulation, consistent with the proteomic analysis. Other pathways were related to the immune system, innate immune system, and IFN signaling, suggesting an antiviral response (Figures 4B and E4B). As has been observed (32), only a small subset of 34 genes and proteins were similarly up- or downregulated in the proteomic and transcriptomic analyses (Table E5).

There were 15 differentially expressed genes in the IFN signaling pathway as displayed in the heatmap (Figure 4C). STRING analysis was applied, and the IFN-induced double-stranded RNA-dependent protein kinase (*PKR*) was determined to be a central node (Figure 4D). When activated, PKR limits viral replication during viral infection. An increase in *PKR*, *RIG-I* (retinoic acid–inducible gene I), *IFNAR1* (type I IFN receptor), and *IFNGR2* (IFN-γ receptor 2) and a decrease in *ARIH1* (ariadne RBR E3 ubiquitin protein ligase 1) are critical in mounting an effective antiviral immune response in neutrophils (33, 34). We chose five IFN-related genes for validation by qPCR and confirmed a PAH neutrophil versus control increase in *PKR*, *RIG-I*, and *IFNAR1* and a decrease in *ARIH1* (Figure 4E). Consistent with the antiviral signature in the transcriptome, VCL and ITGB2 are also implicated in an antiviral response. The upregulation of VCL inhibits retrovirus infection in human cells (35), and a reduction in ITGB2 in monocyte-derived macrophages results in impaired integrity of HIV (36).

### An Increase in HERV-K Envelope Protein in PAH Neutrophils Is Related to the Antiviral Signature and Heightened NE

**Figure 5.**
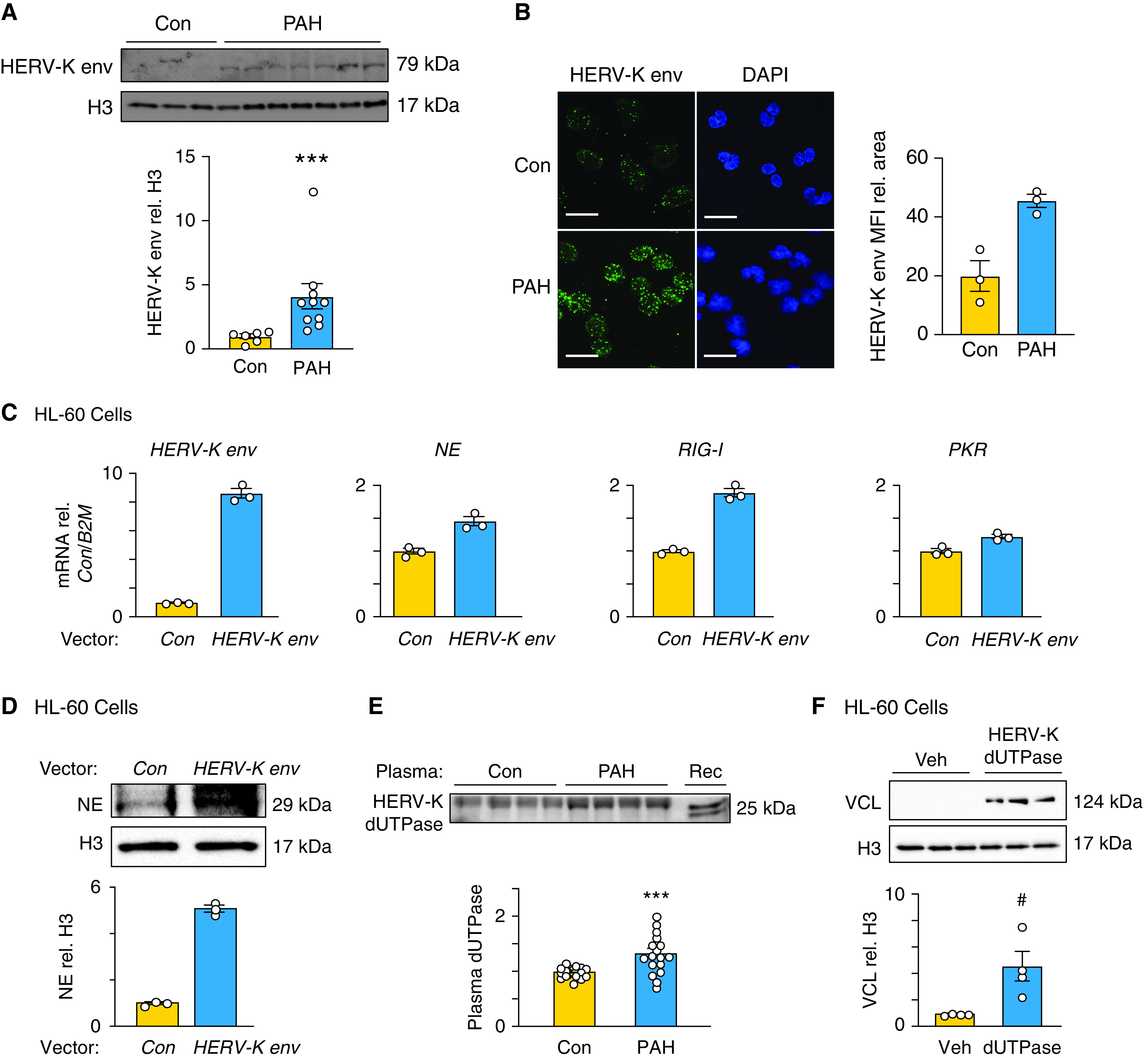
An increase in human endogenous retrovirus (HERV)-K envelope protein in pulmonary arterial hypertension (PAH) neutrophils is related to the antiviral signature and heightened neutrophil elastase (NE). (*A*) Representative western immunoblot analysis and quantification of HERV-K envelope (HERV-K env) relative to histone H3 (H3) in PAH versus control (Con) neutrophils (*n* = 6 Con and *n* = 10 PAH). (*B*) Immunofluorescence microscopy visualization of HERV-K env protein (green) and nuclei by DAPI staining (blue) in Con and PAH neutrophils. Antibodies used for staining are described in the Methods section. A range of z-stack images was collected for image analysis. Mean fluorescence intensity (MFI) and cell area were quantified via ImageJ from three to five randomly selected visual fields (*n* = 3). Scale bar, 20 μm. (*C*–*F*) HL-60 cells were cultured in RPMI complete media and transfected using HL-60 Cell Avalanche transfection reagent per the manufacturer’s protocol. (*C*) RT-qPCR analysis of HERV-K envelope, NE, *RIG-I*, and *PKR* mRNA in HL-60 cells overexpressing HERV-K env (*n* = 3). Raw PCR data, including the comparative cycle threshold calculation, is included in Table E7. (*D*) Representative western immunoblot and quantification of NE relative to H3 in HL-60 cells overexpressing HERV-K env (*n* = 3). (*E*) Western immunoblot analysis and quantification of HERV-K deoxyuridine triphosphate diphosphatase (dUTPase) in plasma collected from Con or patients with PAH. Plasma was depleted of albumin, measured by bicinchoninic acid assay, and run on a gradient gel (*n* = 17). (*F*) Representative western immunoblot analysis and quantification of VCL relative to H3 in dHL-60 cells treated with 10 μg/mL HERV-K dUTPase or Veh (HERV-K dUTPase elution buffer) for 24 hours (*n* = 4). Bars represent mean ± SEM. In *A*, ***P < 0.001 by Mann-Whitney test; in *E*, ***P < 0.001 by unpaired Student’s *t* test; and in *F*, ^#^*P* < 0.05 by Mann-Whitney test.

The antiviral signature, evident from both the transcriptome and the proteome, led us to investigate whether an increase in endogenous retroviral elements might be present in PAH versus control neutrophils. We previously reported upregulation of HERV-K dUTPase in circulating monocytes of patients with PAH and an increase in both the HERV-K dUTPase and the envelope protein in perivascular macrophages in PAH lung tissue sections (37). We assessed HERV-K proteins in PAH versus control neutrophils and found an increase in the HERV-K envelope protein, but not in HERV-K dUTPase (Figures 5A and E5A), by western immunoblot analysis and consistent with an increase by confocal microscopic analysis (Figure 5B). HERV-K peptides were not included in the proteomic database, and HERV-K mRNA may not have been expressed in mature neutrophils, accounting for its absence from the transcriptomic analyses.

To determine whether the increase in HERV-K envelope was responsible for heighted NE and VCL as well as IFN signaling, HL-60 (a neutrophil promyelocytic cell line) was transfected with a HERV-K envelope vector or a control green fluorescent protein vector, and gene expression was analyzed by qPCR (Figure 5C). Overexpression of the HERV-K envelope resulted in a reproducible increase in IFN genes *RIG-I* and *PKR* as well as NE mRNA (Figure 5C) and protein (Figure 5D). There was, however, no significant elevation in VCL concentrations (Figure E5B).

Although we did not observe a difference in PAH neutrophil HERV-K dUTPase (Figure E5E), monocytes can release HERV-K dUTPase, and circulating concentrations of this retroviral protein were increased in PAH versus control plasma samples (Figure 5E). We added the recombinant form of HERV-K dUTPase to differentiated HL-60 cells and observed a reproducible increase in VCL (Figures 5F and E5B). HERV-K dUTPase did not induce an elevation in *PKR* or *RIG-1* mRNA or in NE protein (Figures E5C and E5D). The dose of dUTPase chosen was based on previous studies by our group showing the impact of dUTPase on cytokine release (37) and EndMT (38). We then determined whether the increase in VCL could explain the increase in adhesion and the reduction in migration. Indeed, reducing VCL by siRNA (Figure E6A) completely suppressed the increase in adhesion of dHL-60 cells in response to HERV-K dUTPase (Figure E6B). Migration of dHL-60 cells was neither increased by HERV-K dUTPase nor suppressed by VCL siRNA (Figure E6C).

### CD66b PAH EVs Display Increased NE and Elevated HERV-K Envelope

**Figure 6.**
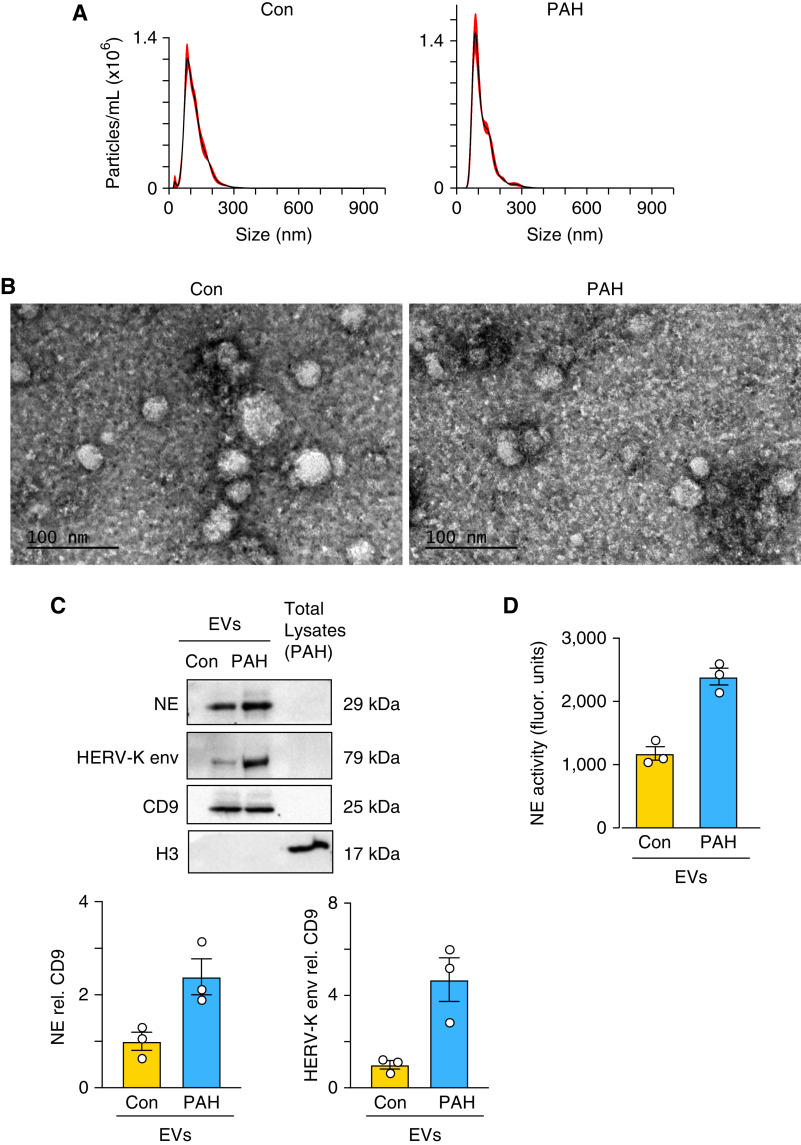
CD66b pulmonary arterial hypertension (PAH) extracellular vesicles (EVs) display increased neutrophil elastase (NE) and elevated human endogenous retrovirus (HERV)-K envelope. EVs were isolated from plasma of 17 healthy donor controls (Con) and 17 patients with PAH using the ExoTIC device described in the Methods section. CD66b-positive neutrophil EVs were pulled down using anti-CD66b beads from pooled EVs of Con and PAH pooled plasma. (*A*) Size distribution of the pooled EVs, determined using NanoSight. (*B*) Representative transmission electron microscopy (TEM) images of CD66b-positive neutrophil EVs derived from pooled plasma of PAH versus Con patients. Scale bar, 100 nm. (*C*) Western immunoblot analysis and quantification of NE and HERV-K envelope from the same number of pooled PAH versus Con neutrophil EVs relative to the EV marker CD9. H3 from PAH neutrophil total lysate was used as a negative control and represented the lack of enrichment of NE and HERV-K with equivalent protein loading. (*D*) NE activity in the same number of PAH versus Con EVs after 120-minute incubation. NE was assessed by the production of BODIPY FL–labeled fluorescent elastin fragments from self-quenching BODIPY FL–conjugated bovine neck ligament elastin. Bars represent mean ± SEM; *n* = 3 technical replicates of the pooled EVs.

Transport of increased NE via neutrophil EVs was observed in bronchial secretions from patients with COPD, and these EVs caused COPD-like changes in mice (18). A CD66b antibody was used to purify neutrophil-specific EVs from PAH and control plasma after total EV isolation using a nanofiltration-based EV isolation tool, EV Total Isolation Chip (39). EV size was confirmed by NanoSight (Figure 6A) and by transmission electron microscopy (Figure 6B). However, the neutrophil supernatant after depletion of CD66b EVs also expressed approximately 50% of the elastase activity seen in the EVs with a relative increase in PAH versus control (Figure E7A), suggesting that only about half of the elastase released is in neutrophil EVs. On the one hand, approximately 50% of total EVs in the plasma were CD66b, but neither total EVs nor CD66b EVs or non-CD66b EVs were increased in PAH versus control plasma (Figure E7B). On the other hand, pooled PAH EVs exhibited an approximately twofold increase in NE protein, assessed by western immunoblot analysis (Figure 6C) and by activity (Figure 6D), as well as a fourfold increase in HERV-K envelope (Figure 6C). Moreover, EV elastase and HERV-K envelope were primarily in the neutrophil (CD66b) versus nonneutrophil (non-CD66b) EVs (Figure E7C).

### CD66b PAH EVs Cause Pulmonary Hypertension in a Mouse Model

**Figure 7.**
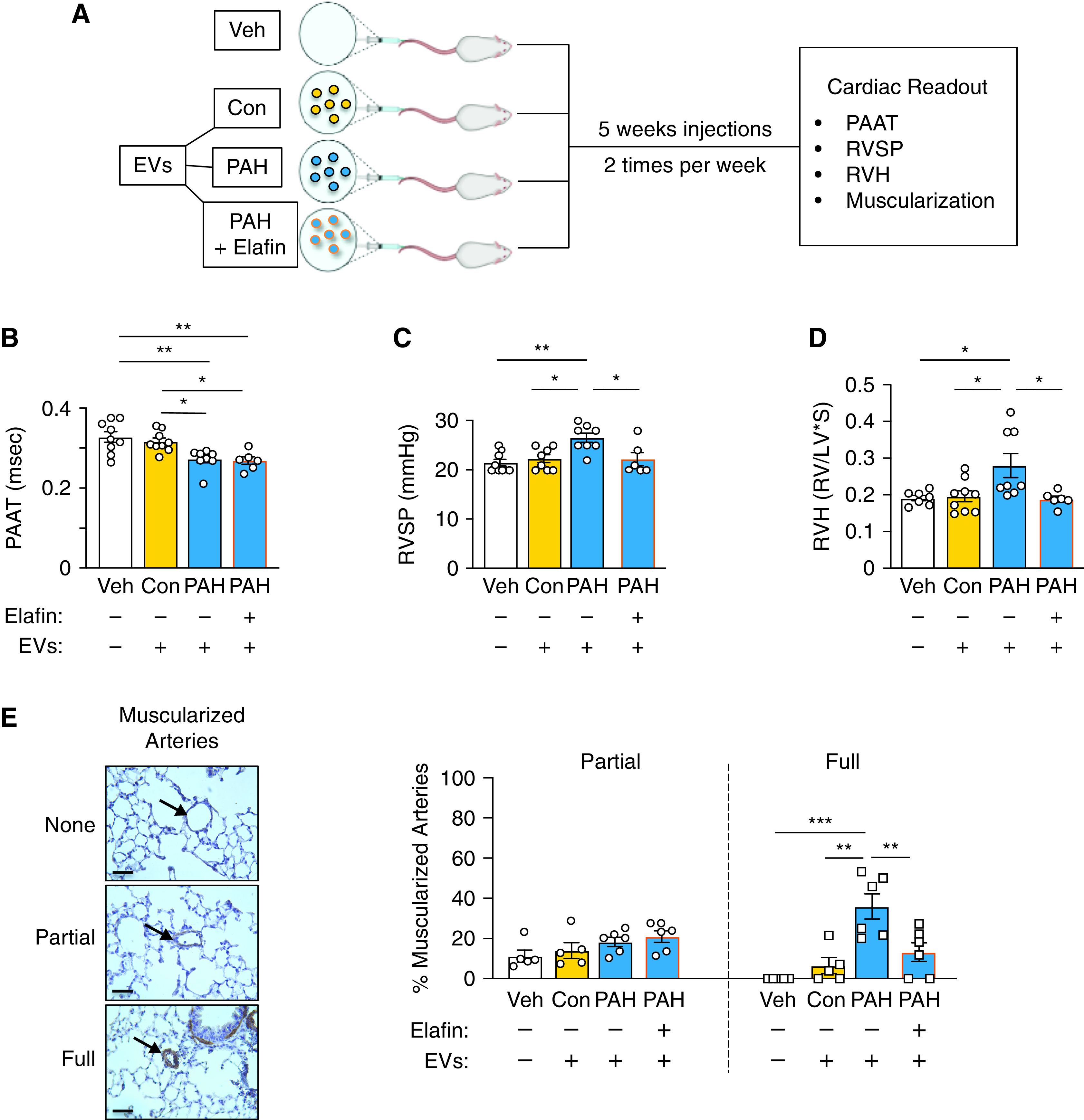
CD66b extracellular vesicles (EVs) cause pulmonary hypertension in a mouse model. CD66b EVs were isolated from plasma of patients with pulmonary arterial hypertension (PAH) or healthy controls (Con). Adult male mice (8 wk) were injected with PAH versus Con plasma EVs (1.63 × 10^7^) at a volume of 100 μl. The PAH plasma EVs were preincubated for 30 minutes with either elafin (0.02 mg/kg mouse weight in a volume of 4–6 μl from a 100 μg/ml stock solution) and/or an equivalent volume of vehicle (phosphate-buffered saline). In a separate experiment, we confirmed that the addition of elafin did not change the number of EVs (PAH EVs, 2.72 × 10^8^ particles/ml; PAH elafin EVs, 2.75 × 10^8^ particles/ml). The EV suspensions were then injected twice per week for 5 weeks. Hemodynamic function was evaluated 2 days after the last injection. (*A*) Illustration of murine study protocol. (*B*) Pulmonary artery acceleration time (PAAT). (*C*) Right ventricular systolic pressure (RVSP). (*D*) Right ventricular hypertrophy (RVH). (*E*) Microscopic images of lung sections of the mice, labeled for α-smooth muscle actin (brown; smooth muscle cell marker) showing examples of nonmuscular, partially muscular, and fully muscularized vessels. Scale bar, 40 μm. In *B*–*E*, bars represent mean ± SEM. *P < 0.05, **P < 0.01, ***P < 0.001 by one-way ANOVA followed by Dunnett’s posttest comparing each group mean with the control group.

Neutrophil EVs isolated from pooled PAH and control plasma were administered by tail vein injection twice per week for 5 weeks to adult male mice (Figure 7A) according to a protocol similar to that used by other investigators to induce COPD in mice (18). The use of pooled plasma minimized the variability from patient to patient but allowed variability in the response of the mice. One mouse in the PAH cohort died before assessment. Experimental pulmonary hypertension was evident after injection of PAH but not control neutrophil EVs, judged by decreased pulmonary artery acceleration time (Figure 7B) and increased right ventricular systolic pressure, right ventricular hypertrophy, and peripheral pulmonary arterial muscularization (Figures 7B–7E). Pretreatment of the EVs with the NE inhibitor and antiviral agent elafin prevented the increase in right ventricular systolic pressure and right ventricular hypertrophy (Figures 7C and 7D) as well as the increased muscularization of the pulmonary arteries (Figure 7E). Cardiac output, left ventricular ejection fraction (Figure E8A), and the relative proportions of neutrophils, monocytes, lymphocytes, and eosinophils (Figure E8B) were not affected by the EV treatment.

## Discussion

**Figure 8.**
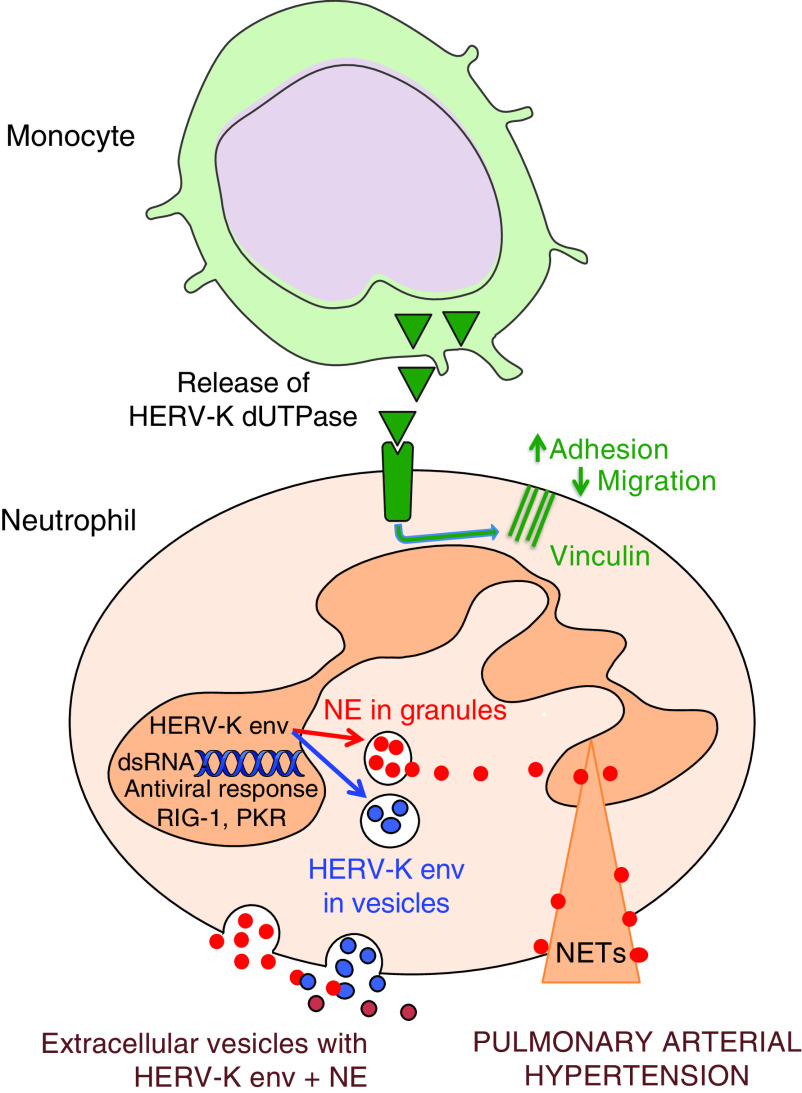
Schematic representation of pulmonary arterial hypertension (PAH) neutrophil. The secretion of human endogenous retrovirus (HERV)-K deoxyuridine triphosphate diphosphatase (dUTPase) from monocytes results in the upregulation of vinculin, thereby increasing neutrophil adhesion and reducing migration. An increase in HERV-K envelope (env) in the neutrophil is likely via double-stranded (ds) RNA required for the IFN response and heightened neutrophil elastase. The increase in neutrophil elastase (NE) promotes neutrophil extracellular traps (NETs). Elastase released from granules associates with HERV-K env in EVs, causing pathologic features of PAH.

Despite many advances in our understanding of the sequelae of chronic inflammation related to PAH pathology, the role of neutrophils has remained elusive. Here, we provide evidence of neutrophil dysfunction in PAH that we link to an antiviral response that results in adverse remodeling of the pulmonary vasculature. PAH neutrophils produce an increase in NE, leading to a propensity to form NETs, and are characterized by increased adhesion and reduced transendothelial migration attributed to elevated concentrations of VCL (35, 36). Augmentation of the HERV-K envelope in PAH neutrophils can explain the increase in elastase and in IFN genes, whereas a heightened concentration of circulating HERV-K dUTPase can induce VCL. EVs released from PAH neutrophils contain increased NE and HERV-K envelope and cause pulmonary hypertension in mice, mitigated by the elastase inhibitor elafin (summary schema in Figure 8).

Our previous studies attributed the increase of NE in PAH to its production by pulmonary arterial SMC in patients and experimental models (40). Here we demonstrate that PAH neutrophils also have heightened NE, which markedly increases the propensity for elastase-mediated tissue damage, in part as a result of heightened predisposition to NET formation previously described in PAH tissues (12, 17, 41) and in part related to NE released in EVs (18). Although NET markers (DNA, myeloperoxidase, and citrullinated histone H3) were previously identified in PAH lung tissue (42), and although NE on NETs is shielded from natural circulating large-molecule inhibitors such as alpha-1 antitrypsin (41), our studies now provide evidence that small protein inhibitors such as elafin can block NE activity and NET formation. They also raise the question whether the success of elafin in previous studies in the Sugen/hypoxia rat model (10) is related to its ability to block neutrophil-derived NE in EVs.

PAH neutrophils are further characterized by increased adhesion and reduced migration that we relate to an increase in neutrophil VCL previously shown to be responsible for these abnormalities (29). Furthermore, VCL suppresses ITGB2 (43), consistent with reduced neutrophil emigration in ITGB2-null mice (31).

A PAH neutrophil antiviral signature consistent with an IFN innate immune response (44) is related to increased NE and VCL. PAH neutrophils express high amounts of endogenous retrovirus HERV-K, consistent with our previous observation in PAH monocytes and macrophages (37). HERVs are noninfectious remnants of ancient viral infections incorporated in our genome. They are highly expressed in embryonic stem cells as part of an innate immune response but undergo silencing in differentiated cells in response to extensive methylation of regulatory elements (45). Expansion of retroviral RNA sequences with production of double-stranded RNA and proteins can occur as part of an innate defense mechanism (46) in response to systemic and environmental cues such as exogenous viruses, including human herpesviruses and HIV (47). Although an increase in HERVs has been described in cancer (48) and in autoimmunity and has been associated with an antiviral IFN response (44), HERV expansion in neutrophils has not been described, and the mechanism for HERV upregulation in disease has been largely elusive. KAP1 (KRAB-associated protein 1) is a methylase implicated in HERV methylation (49) that is controlled by a bone morphogenetic protein–responsive long noncoding RNA, BORG (bone morphogenetic protein/OP-responsive gene) (50). So, it is possible that with reduced BMPR2 (bone morphogenetic protein receptor 2) function, as occurs in PAH, BORG and KAP1 are reduced, resulting in demethylation and increased expression of HERVs (50).

Overexpression of HERV-K envelope in a neutrophil cell line was sufficient to induce the production of NE. Although the increase in NE likely represents the activation of an innate immune response, future studies will be of interest in delineating the molecular mechanism involved. HERVs can also mediate a chronic IFN response in association with the production of double-stranded RNA (46, 51), and IFN treatment is associated with the development of PAH (52), but IFNs have not previously been linked to elastase production. Our studies showing that HL60 cells transfected with HERV-K plasmid induce elastase mRNA suggest that the increase in elastase may take place when mRNA synthesis of elastase is evident (i.e., at the promyelocyte stage). Whether this occurs in response to a specific transcription factor or to a reduction in a microRNA would be of interest to pursue in future studies.

Infection of monocyte-derived macrophages with HIV led to the upregulation of VCL, and, as a consequence, VCL negatively affected the propagation of the virus (53). Similar to HIV, recombinant HERV-K dUTPase also resulted in an increase in VCL, presumably by a similar mechanism.

Pathologic EVs are recognized in many diseases, including autoimmunity (54), cancer (48), and COPD (18). It is interesting that inhibition of EV formation prevented chronic hypoxia-induced PAH in mice (55), Recent studies support HERV-K delivery in tumor EVs as a mechanism contributing to cancer (48) and NE on EVs as directly related to degradation of airway structure in mice, phenocopying COPD (18, 56). In that model, NE on EVs was susceptible to inhibition by small molecules but evaded large protease inhibitors such as alpha-1 antitrypsin (18). Here we identify an increase in both NE and HERV-K envelope in PAH neutrophil EVs and demonstrate that recombinant elafin, a small 6 kD protein, successfully blocked pulmonary hypertension induced by PAH EVs. Elafin is a potent, naturally occurring NE inhibitor (10) with antiinflammatory and antiviral properties that could explain its effect in preventing the adverse impact of HERV-K transferred in neutrophil PAH EVs, but only if the mice recognize the HERV-K envelope as a molecule similar to their own retroviral element, as they did with HERV-K dUTPase. Elafin is a nuclear factor-κB inhibitor, and nuclear factor-κB activation is associated with the IFN antiviral response (57). Elafin also suppresses viral attachment and transcytosis (19). Taken together, our studies reveal a mechanistic relationship between PAH and an increase in HERV-K mRNA and proteins that reprograms neutrophils by inducing an antiviral response. The production of large amounts of NE released in NETs and in EVs can have local and long-range adverse impacts on vascular cells. These features can be targeted therapeutically by elafin.
